# Poisoning deaths in China, 2006–2016

**DOI:** 10.2471/BLT.17.203943

**Published:** 2018-05-01

**Authors:** Lijun Wang, Yue Wu, Peng Yin, Peixia Cheng, Yunning Liu, David C Schwebel, Jinlei Qi, Peishan Ning, Jiangmei Liu, Xunjie Cheng, Maigeng Zhou, Guoqing Hu

**Affiliations:** a National Center for Chronic and Noncommunicable Disease Control and Prevention, Chinese Center for Disease Control and Prevention, Beijing, China.; b Department of Occupational and Environmental Health, Xiangya School of Public Health, Central South University, Changsha, China.; c Department of Epidemiology and Health Statistics, Xiangya School of Public Health, Central South University, Changsha, China.; d Department of Psychology, University of Alabama at Birmingham, Birmingham, Alabama, United States of America.; Correspondence to Guoqing Hu (email: huguoqing009@gmail.com).

## Abstract

**Objective:**

To provide a comprehensive overview of poisoning mortality patterns in China.

**Methods:**

Using mortality data from the Chinese national disease surveillance points system, we examined trends in poisoning mortality by intent and substance from 2006 to 2016. Differences over time between urban and rural residents among different age groups and across external causes of poisoning were quantified using negative binomial models for males and females separately.

**Results:**

In 2016, there were 4936 poisoning deaths in a sample of 84 060 559 people (5.9 per 100 000 people; 95% confidence interval: 5.6–6.2). Age-adjusted poisoning mortality dropped from 9.2 to 5.4 per 100 000 people between 2006 and 2016. Males, rural residents and older adults consistently had higher poisoning mortality than females, urban residents and children or young adults. Most pesticide-related deaths (34 996 out of 39 813) were suicides among persons older than 15 years, although such suicides decreased between 2006 and 2016 (from 6.1 per 100 000 people to 3.6 for males and from 5.8 to 3.0 for females). In 2016, alcohol caused 29.3% (600/2050) of unintentional poisoning deaths in men aged 25–64 years. During the study period, unintentional fatal drug poisoning by narcotics and psychodysleptics in individuals aged 25–44 years increased from 0.4 per 100 000 people to 0.7 for males and from 0.05 to 0.13 for females.

**Conclusion:**

Despite substantial decreases in mortality, poisoning is still a public health threat in China. This warrants further research to explore causative factors and to develop and implement interventions targeting at-risk populations.

## Résumé

**Objectif:**

Offrir un aperçu détaillé des schémas de mortalité par empoisonnement en Chine.

**Méthodes:**

À partir de données sur la mortalité provenant du système national chinois à points pour la surveillance des maladies, nous avons examiné les tendances de la mortalité par empoisonnement, suivant les intentions et les substances, de 2006 à 2016. Les différences au fil du temps entre résidents urbains et ruraux, de différentes tranches d'âge et pour différentes causes externes d'empoisonnement, ont été quantifiées à l'aide de modèles binomiaux négatifs, en séparant les hommes et les femmes.

**Résultats:**

En 2016, on a compté 4936 empoisonnements mortels sur un échantillon de 84 060 559 personnes (5,9 pour 100 000 habitants; intervalle de confiance de 95%: 5,6–6,2). La mortalité par empoisonnement ajustée en fonction de l'âge est passée de 9,2 à 5,4 pour 100 000 habitants entre 2006 et 2016. Les hommes, les résidents ruraux et les adultes les plus âgés affichaient systématiquement une mortalité par empoisonnement supérieure à celle des femmes, des résidents urbains et des enfants ou jeunes adultes. La plupart des décès dus à des pesticides (34 996 sur 39 813) étaient des suicides de personnes de plus de 15 ans, bien que ce type de suicides ait diminué entre 2006 et 2016 (passant de 6,1 à 3,6 pour 100 000 habitants chez les hommes et de 5,8 à 3,0 chez les femmes). En 2016, l'alcool a causé 29,3% (600/2050) des empoisonnements mortels involontaires chez les hommes de 25 à 64 ans. Durant la période étudiée, les empoisonnements mortels involontaires dus à des narcotiques et des psychodysleptiques chez les personnes âgées de 25 à 44 ans sont passés de 0,4 à 0,7 pour 100 000 habitants chez les hommes et de 0,05 à 0,13 chez les femmes.

**Conclusion:**

En dépit de fortes baisses de la mortalité, les empoisonnements restent une menace pour la santé publique en Chine. Cela justifie de mener des recherches plus poussées afin d’étudier les facteurs qui en sont à l'origine et d’élaborer puis de déployer des interventions axées sur les populations à risque.

## Resumen

**Objetivo:**

Ofrecer una visión general integradora de los patrones de mortalidad por intoxicación en China.

**Métodos:**

Utilizando datos de mortalidad del sistema nacional chino de puntos de vigilancia de enfermedades, examinamos las tendencias en la mortalidad por intoxicación con intención y fundamento de 2006 a 2016. Se cuantificaron las diferencias en el tiempo entre los residentes urbanos y rurales para diferentes grupos por edades y considerando causas externas de intoxicación, utilizando modelos binomiales negativos para hombres y mujeres de forma independiente.

**Resultados:**

En 2016, hubo 4936 muertes por intoxicación en una muestra de 84 060 559 personas (5,9 por cada 100 000 personas; intervalo de confianza del 95%: 5,6–6,2). La mortalidad por intoxicación ajustada por edades disminuyó de 9,2 a 5,4 por cada 100 000 personas entre 2006 y 2016. Los hombres, los habitantes de zonas rurales y los adultos mayores tuvieron una mortalidad superior por intoxicación comparado con las mujeres, los habitantes de zonas urbanas y los niños o adultos jóvenes. La mayoría de las muertes relacionadas con pesticidas (34 996 de 39 813) fueron suicidios entre personas mayores de 15 años, aunque estos suicidios disminuyeron entre 2006 y 2016 (de 6,1 por cada 100 000 personas a 3,6 en los hombres y de 5,8 a 3,0 en las mujeres). En 2016, el alcohol provocó el 29,3% (600/2050) de las muertes por intoxicación no intencionadas en hombres entre las edades de 25 y 64 años. Durante el periodo de estudio, la intoxicación fatal con fármacos no intencionada por narcóticos y psicodislépticos en individuos entre las edades de 25 y 44 años aumentó de 0,4 por cada 100 000 personas a 0,7 en los hombres y de 0,05 a 0,13 en las mujeres.

**Conclusión:**

A pesar de la disminución sustancial en la mortalidad, la intoxicación sigue siendo una amenaza para la salud pública en China. Esto justifica más investigaciones para explorar factores causales y desarrollar e implementar intervenciones orientadas a las poblaciones en riesgo.

## ملخص

**الغرض:**

تقديم نظرة شاملة لأنماط الوفيات الناتجة عن التسمم في الصين.

**الطريقة:**

من خلال ****استخدامنا لبيانات الوفيات من نظام النقاط الوطني الصيني لمراقبة الأمراض، قمنا بفحص النزعات السائدة في حالات وفيات التسمم من حيث القصد والمضمون من عام 2006 إلى عام 2016. وقد تم قياس الاختلافات على مدى فترات من الزمن بين سكان الحضر والريف وذلك ضمن فئات عمرية مختلفة وعبر أسباب خارجية للتسمم باستخدام نماذج ثنائية سلبية للذكور والإناث بشكل منفصل.

النتائج في عام 2016 كانت هناك 4936 حالة وفاة ناتجة عن التسمم في عينة مكونة من 84060559 شخصًا (بواقع 5.9 لكل 100 ألف شخص؛ وبنسبة أرجحية مقدارها 95‏%: 5.6‏–‏6.2). وقد انخفض معدل الوفيات المعدلة حسب العمر والناتجة عن التسمم من 9.2 إلى 5.4 لكل 100 ألف شخص بين عامي 2006 و2016. وقد وُجد أن حالات الوفيات الناتجة عن التسمم مرتفعة بشكل ثابت لدى الذكور وسكان الريف والبالغين الأكبر سنًا مقارنًة بالإناث وسكان الحضر والأطفال أو البالغين الأصغر سنًا. وقد كانت معظم الوفيات المرتبطة بمبيد الآفات (34996 من بين 39813) هي حالات انتحار بين أشخاص تزيد أعمارهم عن 15 عامًا، على الرغم من انخفاض هذه الحالات ما بين عامي 2006 و2016 (من 6.1 لكل 100 ألف شخص إلى 3.6 للذكور ومن 5.8 إلى 3.0 للإناث). وفي عام 2016 تسبب الكحول في نسبة ‏29.3% (600/2050) من حالات الوفيات غير المقصودة الناتجة عن التسمم بين الرجال الذين تتراوح أعمارهم من 25 إلى 64 عامًا. وخلال فترة الدراسة ارتفعت حالات تسمم العقاقير القاتل وغير المقصود بفعل العقاقير المخدرة وعقاقير الهلوسة لدى الأشخاص الذين تتراوح أعمارهم ما بين 25 و44 عامًا من 0.4 لكل 100 ألف شخص إلى 0.7 للذكور ومن 0.05 إلى 0.13 للإناث.

**الاستنتاج:**

بالرغم من الانخفاض الكبير في حالات الوفيات فإن التسمم لازال يمثل تهديدًا للصحة العامة في الصين، الأمر الذي يستدعي ضرورة إجراء المزيد من الأبحاث لاستكشاف العناصر المسببة وتطوير وتنفيذ التدخلات التي تستهدف القطاعات السكانية المعرضة للخطر.

## 摘要

**目的:**

旨在全面综述中国中毒死亡概况。

**方法:**

我们采用《中国疾病监测系统死因监测数据集》，根据意图和物质分类研究了 2006 年至 2016 年间的中毒死亡趋势。我们分别针对男性和女性采用了负二项模型，对不同年龄段城市和农村居民及中毒的外部原因随着时间的差异变化进行了量化。

**结果:**

在 2016 年，84 060 559 人的样本中共有 4936 人中毒死亡（每 100 000 人中有 5.9 人；95% 置信区间：5.6–6.2）。2006 至 2016 年间，年龄标准化中毒死亡率从每 100 000 人中有 9.2 人降至 5.4 人。男性、农村居民和老年人群的中毒死亡率始终高于女性、城市居民和儿童或青壮年人群的中毒死亡率。大部分与杀虫剂有关的自杀死亡（39 813 例中有 34 996 例）发生于 15 岁以上人群，尽管此类自杀在 2006 至 2016 年间有所减少（男性死亡人数从每 100 000 人中由 6.1 人降至 3.6 人，女性死亡人数从 5.8 人降至 3.0 人）。在 2016 年，年龄在 25 岁至 64 岁之间的男性由于酒精而引起事故性中毒死亡的概率为 29.3%（600/2050）。本次研究期间，年龄在 25 岁至 44 岁之间的男性人群发生事故性致命药物（麻醉品和致幻药）中毒死亡的比例从每 100 000 人中有 0.4 人增至 0.7 人，女性从 0.05 人增至 0.13 人。

**结论:**

尽管由于中毒导致的死亡率已显著降低，但在中国，中毒仍旧会对公共健康造成威胁。有必要开展进一步的研究以探索诱发因素并制定和实施针对有中毒死亡风险人群的干预措施。

## Резюме

**Цель:**

Предоставить всесторонний обзор показателей смертности в результате отравления в Китае.

**Методы:**

Используя данные о смертности, полученные из национальной системы эпиднадзора Китая, авторы изучили тенденции смертности в результате отравления по фактору преднамеренности и по отравляющему веществу с 2006 по 2016 год. Динамика различий между городскими и сельскими жителями среди разных возрастных групп, а также по внешним причинам отравления определялась количественно с использованием отрицательных биномиальных моделей отдельно для мужчин и женщин.

**Результаты:**

В 2016 году было зарегистрировано 4936 случаев летального исхода в результате отравления в выборке из 84 060 559 человек (5,9 на 100 000 человек, 95%-й ДИ: 5,6–6,2). В период с 2006 по 2016 год стандартизированный по возрасту уровень смертности в результате отравления снизился с 9,2 до 5,4 на 100 000 человек. Мужчины, сельские жители и пожилые люди равным образом имели более высокий уровень смертности в результате отравления, чем женщины, жители городов, дети или молодые люди. Причиной большинства случаев летального исхода в результате отравления пестицидами (34 996 из 39 813) было самоубийство среди лиц старше 15 лет, хотя количество таких самоубийств снизилось в период между 2006 и 2016 годами (с 6,1 на 100 000 человек до 3,6 для мужчин и с 5,8 до 3,0 для женщин). В 2016 году алкоголь был причиной 29,3% случаев (600/2050) непреднамеренной смерти в результате отравления у мужчин в возрасте 25–64 лет. В течение периода исследования количество случаев непреднамеренного отравления лекарствами и наркотическими веществами с летальным исходом у лиц в возрасте 25–44 лет увеличилось с 0,4 на 100 000 человек до 0,7 для мужчин и с 0,05 до 0,13 для женщин.

**Вывод:**

Несмотря на значительное снижение смертности, отравление по-прежнему представляет угрозу для общественного здравоохранения в Китае. Это требует дальнейших исследований для изучения причинно-следственных факторов, а также для разработки и реализации мероприятий, ориентированных на группы риска.

## Introduction

According to the Global Burden of Disease 2015 update, approximately 86 353 people died from unintentional poisonings worldwide in 2015, with 78 054 (90%) deaths occurring in low- and middle-income countries.^1^ However, if the global estimates of the numbers of intentional poisonings were freely accessible, the poisoning mortality numbers would be higher.^1^

Despite the implications for public health and the impact described in high-income countries,^2^^–^^10^ poisonings in low- and middle-income countries are poorly understood. We are aware of just two published studies on the epidemiology of poisoning at the national level in low- and middle-income countries: from Fiji and the Islamic Republic of Iran.^11^^,^^12^

As the most populated country in the world, China had 16 179 unintentional poisoning deaths in 2016, 31% of the world’s total of 52 077.^1^ Available knowledge about poisoning incidents in China is scattered. A study using Global Burden of Disease 2015 update data examined trends in unintentional poisoning deaths.^13^ The authors reported a substantial reduction in unintentional poisoning mortality from 1990 to 2015. A second study used the Chinese disease surveillance points system data and reported decreases in suicide by poisoning from 2006 to 2013.^14^ The authors also reported that suicide by pesticide poisoning was the leading method of suicide. Other published studies have examined poisoning patterns within one hospital catchment area or one province^15^^,^^16^ or focused on the epidemiology of a single cause of poisoning, such as pesticide poisoning.^17^

We found no recent, comprehensive published studies of the epidemiology of fatal poisonings in China. To address these gaps, we used national disease surveillance data to examine changes in poisoning mortality from 2006 to 2016 by location (urban or rural), age group, intent and type of substance. Analyses were conducted separately for males and females.

## Methods

### Data source

We designed a population-based longitudinal study based on the data from the national disease surveillance points system, initiated in 1978 by the Chinese government. The surveillance system has undergone three major improvements since 1978. First, it was expanded from 145 to 161 points in 2004–2006, yielding coverage of about 73 million residents.^18^ Second, a web-based approach was introduced to report deaths in 2008, a development that greatly improved the timeliness of data reporting.^18^ Third, the Chinese government combined the system with the national vital registration system in 2013, creating a data collection system from 605 surveillance points.^18^ The population of China in the most recent census in 2010 was around 1332 million (682 million males and 650 million females).

The data for the disease surveillance points system are collected using a standard protocol by trained persons.^18^ Trained staff members oversee data collection of all deaths occurring in the hospital. For deaths occurring outside the hospital, village health workers or community hospital professionals use verbal autopsy strategies to collect the relevant data. Local centres for disease prevention and control report all data to their next-level office (from county to prefectural to provincial to national) and routine quality checks are conducted by coders at each centre. Quality checks assess completeness, coding and internal logic across items reported on death certificates.^18^ Any unqualified reports that are detected are corrected at each surveillance point through a review of detailed medical records or repeated verbal autopsies.^19^ Additionally, a routine national sample survey is conducted every 3 years at all surveillance locations to adjust for any under-reporting overlooked by daily quality checks.^20^

We extracted the numbers of poisoning deaths and mortality per 100 000 people from 2006 to 2016. The present study reports mortality rates for 2006–2013 from 161 surveillance points and for 2014–2016 from 605 surveillance points.

This analysis was approved by the ethics committee of Xiangya School of Public Health, Central South University. Data were de-identified.

### Classification of poisoning

Using the *International statistical classification of diseases and related health problems, 10th revision* (ICD–10), poisoning is divided into four intent categories: unintentional (codes X40‒X49), suicide (X60‒X69), homicide (X85‒X90) and undetermined (Y10‒Y19).^21^ Poisoning is also classified into five groups by type of substance: drug (codes X40‒X44, X60‒X64, X85, Y10‒Y14), alcohol (X45, X65, Y15), pesticide (X48, X68, X87, Y18), other gases/vapours (X47, X67, Y17) and all others (X46, X49, X66, X69, X86, X88‒X90, Y16, Y19). We analysed drug poisoning in nine categories of intent and substance: opioid analgesics, antipyretics and antirheumatics (unintentional: code X40; suicide: X60); antiepileptics, sedative-hypnotics, antiparkinsonism and psychotropic drugs (unintentional: X41; suicide: X61); narcotics and psychodysleptics [hallucinogens] (unintentional: X42; suicide: X62); other drugs (unintentional: X43, X44; suicide: X63, X64); and drug poisoning with other intents (X85, Y10‒Y14).

### Data analysis

We considered three demographic factors in the analyses: location (urban or rural), age group and year. Based on preliminary analysis and human development theory, we classified age into six groups: 0–4, 5–14, 15–24, 25–44, 45–64 and ≥ 65 years.

We calculated age-adjusted mortality rates and 95% confidence intervals (CI), using the 2010 population of China as the reference. To overcome over-dispersion of count data^22^ we ran univariate negative binomial regression to quantify poisoning mortality changes between 2006 and 2016, using percentage change in mortality rate and its 95% CI. We first calculated the mortality rate ratio by dividing the mortality rate for 2016 with the corresponding rate of 2006, and then calculated the percentage change as: (mortality rate ratio−1) × 100.

We found that the location-specific analysis showed similar changes to that in overall poisoning mortality and that multistrata subgroup analysis yielded unreliable subgroup mortality rates for many groups due to the small numerators (deaths < 20).^23^ We therefore analysed age-specific fatal poisonings by intent and type of substance.

We conducted all analyses separately for males and females. Statistical analyses were completed using Stata version 12.1 (StataCorp LLC, College Station, United States of America; USA).

## Results

### Overall trends

Between 2006 and 2016, 67 713 poisoning deaths were reported to the disease surveillance points system (Table 1 and Table 2; available at: http://www.who.int/bulletin/volumes/96/5/17-203943). Of those, 41 378 (61%) were among males and 51 761 (76%) were among people living in rural areas.

**Table 1:** Number of poisoning deaths by location, age group, type of substance and intent in China, 2006–2016: males

Variable	No. of deaths by year										
	2006	2007	2008	2009	2010	2011	2012	2013	2014	2015	2016
**All**	3 895	3 979	4 314	3 996	3 964	3 826	3 659	3 620	3 467	3 483	3 175
**Location**											
Urban	904	946	964	967	948	875	813	844	875	883	822
Rural	2 991	3 033	3 350	3 029	3 016	2 951	2 846	2 776	2 592	2 600	2 353
**Age group**											
0–4 years	29	20	30	36	37	45	34	39	23	24	27
5–14 years	59	69	59	48	54	52	35	42	33	32	34
15–24 years	300	264	289	286	308	289	236	217	196	187	144
25–44 years	1 100	1 149	1 264	1 145	1 137	1 108	1 020	1 000	1 003	969	796
45–64 years	1 249	1 296	1 453	1 329	1 312	1 306	1 267	1 311	1 226	1 312	1 254
≥ 65 years	1 158	1 181	1 219	1 152	1 116	1 026	1 067	1 011	986	959	920
**External cause**											
Drug^a^	363	436	479	421	473	475	420	438	473	394	307
Alcohol	721	687	777	759	807	753	771	720	719	780	711
Pesticides	2 120	2 200	2 293	2 105	2 002	1 942	1 898	1 829	1 740	1 720	1 516
Other gases/vapours	454	436	575	534	533	504	449	516	428	499	552
Other poisoning	237	220	190	177	149	152	121	117	107	90	89
**Intent**											
Unintentional	1 412	1 420	1 732	1 750	1 810	1 863	1 727	1 780	1 705	1 804	1 652
Suicide	2 278	2 380	2 302	2 078	1 956	1 823	1 809	1 706	1 664	1 602	1 452
Undetermined	200	168	269	155	190	128	116	126	91	74	69

^a^ Drugs include nonopioid analgesic, antipyretic and antirheumatic drugs; sedative-hypnotic, antiparkinsonism and psychotropic drugs and narcotics and psychodysleptics [hallucinogens].

**Table 2:** Number of poisoning deaths by location, age group, type of substance and intent in China, 2006–2016: females

Variable	No. of deaths by year										
	2006	2007	2008	2009	2010	2011	2012	2013	2014	2015	2016
**All**	2 737	2 773	2 975	2 687	2 556	2 468	2 358	2 089	1 987	1 944	1 761
**Location**											
Urban	650	604	654	613	570	556	520	481	495	496	472
Rural	2 087	2 169	2 321	2 074	1 986	1 912	1 838	1 608	1 492	1 448	1 289
**Age group**											
0–4 years	17	17	46	25	22	39	20	18	18	18	15
5–14 years	44	42	41	31	38	49	43	30	24	17	16
15–24 years	243	239	281	230	266	225	194	150	125	105	72
25–44 years	815	821	852	779	631	649	542	493	401	393	314
45–64 years	726	734	793	698	711	672	727	621	571	586	559
≥ 65 years	892	920	962	924	888	834	832	777	848	825	785
**External cause**											
Drug^a^	226	267	299	231	221	204	187	175	177	194	135
Alcohol	33	37	50	33	42	45	40	27	25	49	39
Pesticides	2 025	2 061	2 149	2 033	1 877	1 725	1 689	1 538	1 409	1 360	1 209
Other gases/vapours	260	231	337	278	317	374	336	279	301	273	329
Other poisoning	193	177	140	112	99	120	106	70	75	68	49
**Intent**											
Unintentional	522	533	684	658	684	737	700	596	607	593	575
Suicide	2 095	2 178	2 183	1 951	1 806	1 668	1 611	1 449	1 344	1 317	1 152
Undetermined	105	54	103	68	54	57	43	40	28	31	33

^a^ Drugs include nonopioid analgesic, antipyretic and antirheumatic drugs; sedative-hypnotic, antiparkinsonism and psychotropic drugs and narcotics and psychodysleptics [hallucinogens].

In 2016, there were 4936 poisoning deaths in the Chinese disease surveillance sample of 84 060 559 (3175 of 42 752 062 among males and 1761 of 41 308 497 among females). The overall crude poisoning mortality was 5.9 (95% CI: 5.6–6.2) per 100 000 people: 7.4 (95% CI: 7.2–7.7) and 4.3 (95% CI: 4.1–4.5) per 100 000 in males and females, respectively.

Age-adjusted mortality fell from 9.2 to 5.4 per 100 000 people between 2006 and 2016. For both sexes we found a trend of a slight increase in age-adjusted mortality from 2006 to 2008 and then a gradual decrease from 2009 to 2016 (Table 3; Table 4). Males consistently had higher poisoning mortality than females across the study period (male to female ratio range: 1.4–1.8). Poisoning mortality per 100 000 people decreased by 36% (from 10.7 to 6.9) for males and 50% (from 7.6 to 3.8) for females between 2006 and 2016.

**Table 3:** Age-adjusted poisoning mortality per 100 000 people by location, age group, type of substance and intent in China, 2006–2016: males

Variable	Mortality per 100 000 people (95% CI) by year											% change in rate (95% CI)^a^
	2006	2007	2008	2009	2010	2011	2012	2013	2014	2015	2016	
**All**	10.7 (10.4 to 11.0)	10.7 (10.3 to 11.0)	11.3 (11.0 to 11.6)	10.3 (10.0 to 10.6)	10.1 (9.8 to 10.4)	9.1 (8.8 to 9.4)	8.6 (8.3 to 8.8)	8.3 (8.0 to 8.5)	7.9 (7.6 to 8.2)	7.9 (7.7 to 8.2)	6.9 (6.7 to 7.2)	−35 (−38 to −32)
**Location**												
Urban	6.3 (5.9 to 6.7)	6.4 (6.0 to 6.8)	6.3 (5.9 to 6.8)	6.3 (5.9 to 6.7)	6.1 (5.7 to 6.5)	4.6 (4.3 to 4.9)	4.3 (4.0 to 4.6)	4.3 (4.0 to 4.6)	4.3 (4.0 to 4.7)	4.4 (4.1 to 4.7)	3.9 (3.6 to 4.1)	−39 (−44 to −33)
Rural	13.7 (13.2 to 14.1)	13.5 (13.1 to 14.0)	14.7 (14.2 to 15.2)	13.1 (12.6 to 13.5)	12.8 (12.3 to 13.2)	12.7 (12.2 to 13.1)	11.9 (11.4 to 12.4)	11.5 (11.0 to 11.9)	10.9 (10.5 to 11.4)	11.0 (10.5 to 11.4)	9.6 (9.2 to 10.0)	−30 (−33 to −26)
**Age group**												
0–4 years	1.2 (0.8 to 1.7)	0.8 (0.5 to 1.2)	1.2 (0.8 to 1.7)	1.5 (1.0 to 1.9)	1.5 (1.0 to 1.9)	1.9 (1.4 to 2.5)	1.4 (0.9 to 1.9)	1.6 (1.1 to 2.1)	0.9 (0.5 to 1.3)	1.0 (0.6 to 1.4)	1.1 (0.7 to 1.5)	−12 (−48 to 49)
5–14 years	1.2 (0.9 to 1.5)	1.4 (1.1 to 1.7)	1.2 (0.9 to 1.5)	1.0 (0.7 to 1.3)	1.1 (0.8 to 1.4)	1.2 (0.9 to 1.5)	0.8 (0.5 to 1.0)	0.9 (0.7 to 1.2)	0.7 (0.5 to 1.0)	0.7 (0.5 to 1.0)	0.8 (0.5 to 1.0)	−37 (−59 to −4)
15–24 years	4.4 (3.9 to 4.9)	3.8 (3.3 to 4.3)	4.2 (3.7 to 4.7)	4.2 (3.7 to 4.7)	4.6 (4.1 to 5.1)	4.1 (3.7 to 4.6)	3.5 (3.0 to 3.9)	3.2 (2.8 to 3.7)	3.0 (2.6 to 3.4)	2.9 (2.5 to 3.3)	2.2 (1.8 to 2.5)	−51 (−60 to −40)
25–44 years	8.6 (8.1 to 9.1)	8.7 (8.2 to 9.2)	9.6 (9.0 to 10.1)	8.8 (8.3 to 9.3)	8.9 (8.4 to 9.4)	8.1 (7.6 to 8.6)	7.5 (7.1 to 8.0)	7.6 (7.1 to 8.1)	7.8 (7.3 to 8.2)	7.5 (7.1 to 8.0)	6.1 (5.7 to 6.5)	−29 (−35 to −22)
45–64 years	14.6 (13.8 to 15.4)	14.9 (14.0 to 15.7)	16.0 (15.2 to 16.8)	13.8 (13.0 to 14.5)	13.1 (12.4 to 13.8)	12.2 (11.5 to 12.9)	11.6 (10.9 to 12.2)	11.3 (10.7 to 11.9)	10.4 (9.8 to 11.0)	11.2 (10.6 to 11.8)	10.7 (10.1 to 11.3)	−26 (−32 to −20)
≥ 65 years	40.3 (38.0 to 42.6)	39.6 (37.4 to 41.9)	40.1 (37.8 to 42.3)	37.4 (35.2 to 39.5)	35.7 (33.6 to 37.8)	29.9 (28.0 to 31.7)	29.9 (28.1 to 31.7)	27.5 (25.8 to 29.2)	26.0 (24.4 to 27.6)	25.2 (23.6 to 26.8)	21.5 (20.1 to 22.8)	−47 (−51 to −42)
**External cause**												
Drug^b^	1.0 (0.9 to 1.1)	1.1 (1.1 to 1.2)	1.2 (1.2 to 1.3)	1.1 (1.0 to 1.1)	1.2 (1.1 to 1.3)	1.1 (1.1 to 1.2)	1.0 (0.9 to 1.1)	1.1 (1.0 to 1.1)	1.1 (1.1 to 1.2)	0.9 (0.9 to 1.0)	0.7 (0.6 to 0.8)	−28 (−38 to −17)
Alcohol	2.0 (1.9 to 2.1)	1.8 (1.7 to 1.9)	2.0 (1.9 to 2.1)	1.9 (1.8 to 2.0)	2.0 (1.9 to 2.1)	1.8 (1.7 to 1.9)	1.8 (1.7 to 1.9)	1.6 (1.5 to 1.7)	1.6 (1.5 to 1.7)	1.8 (1.7 to 1.9)	1.6 (1.5 to 1.7)	−21 (−29 to −12)
Pesticides	5.9 (5.7 to 6.0)	5.9 (5.8 to 6.1)	6.1 (5.9 to 6.2)	5.5 (5.3 to 5.6)	5.1 (5.0 to 5.3)	4.6 (4.5 to 4.8)	4.4 (4.3 to 4.6)	4.2 (4.0 to 4.3)	3.9 (3.8 to 4.0)	3.9 (3.7 to 4.0)	3.3 (3.1 to 3.4)	−45 (−48 to −41)
Other gases/vapours	1.2 (1.2 to 1.3)	1.2 (1.1 to 1.2)	1.5 (1.4 to 1.6)	1.4 (1.3 to 1.4)	1.4 (1.3 to 1.4)	1.2 (1.1 to 1.3)	1.1 (1.0 to 1.1)	1.2 (1.1 to 1.3)	1.0 (0.9 to 1.1)	1.2 (1.1 to 1.2)	1.2 (1.1 to 1.3)	−2 (−13 to 11)
Other poisoning	0.7 (0.6 to 0.7)	0.6 (0.5 to 0.6)	0.5 (0.4 to 0.6)	0.5 (0.4 to 0.5)	0.4 (0.3 to 0.4)	0.4 (0.3 to 0.4)	0.3 (0.3 to 0.3)	0.3 (0.2 to 0.3)	0.3 (0.2 to 0.3)	0.2 (0.2 to 0.2)	0.2 (0.2 to 0.2)	−70 (−77 to −61)
**Intent**												
Unintentional	3.8 (3.6 to 4.0)	3.8 (3.6 to 4.0)	4.5 (4.3 to 4.7)	4.5 (4.3 to 4.7)	4.6 (4.4 to 4.8)	4.4 (4.2 to 4.6)	4.0 (3.8 to 4.2)	4.1 (3.9 to 4.3)	3.9 (3.7 to 4.1)	4.1 (3.9 to 4.3)	3.6 (3.4 to 3.8)	−5 (−12 to 2)
Suicide	6.3 (6.1 to 6.6)	6.4 (6.2 to 6.7)	6.1 (5.8 to 6.3)	5.4 (5.2 to 5.6)	5.0 (4.8 to 5.2)	4.3 (4.1 to 4.5)	4.2 (4.0 to 4.4)	3.9 (3.7 to 4.1)	3.8 (3.6 to 3.9)	3.6 (3.4 to 3.8)	3.1 (2.9 to 3.3)	−50 (−54 to −47)
Undetermined	0.5 (0.5 to 0.6)	0.4 (0.4 to 0.5)	0.7 (0.6 to 0.8)	0.4 (0.3 to 0.5)	0.5 (0.4 to 0.5)	0.3 (0.2 to 0.4)	0.3 (0.2 to 0.3)	0.3 (0.2 to 0.3)	0.2 (0.2 to 0.3)	0.2 (0.1 to 0.2)	0.2 (0.1 to 0.2)	−71 (−78 to −62)

CI: confidence interval.

^a^ Percentage change in mortality and its 95% CI was calculated as: (mortality rate ratio−1) × 100.

^b^ Drugs include nonopioid analgesic, antipyretic and antirheumatic drugs; sedative-hypnotic, antiparkinsonism and psychotropic drugs and narcotics and psychodysleptics [hallucinogens].

Note: Mortality for overall and subgroup poisoning rates (excluding the six age groups) were age-adjusted using the population of China in 2010 (the most recent census).

**Table 4:** Age-adjusted poisoning mortality per 100 000 people by location, age group, type of substance and intent in China, 2006–2016: females

Variable	Mortality per 100 000 people (95% CI) by year											% change in rate (95% CI)^a^
	2006	2007	2008	2009	2010	2011	2012	2013	2014	2015	2016	
**All**	7.6 (7.3 to 7.9)	7.7 (7.4 to 8.0)	8.1 (7.8 to 8.4)	7.2 (6.9 to 7.5)	6.8 (6.5 to 7.0)	6.0 (5.8 to 6.3)	5.7 (5.5 to 5.9)	4.9 (4.7 to 5.1)	4.6 (4.4 to 4.8)	4.5 (4.3 to 4.7)	3.8 (3.6 to 4.0)	−50 (−53 to −46)
**Location**												
Urban	4.6 (4.2 to 4.9)	4.2 (3.9 to 4.6)	4.5 (4.1 to 4.8)	4.1 (3.8 to 4.5)	3.9 (3.5 to 4.2)	3.1 (2.9 to 3.4)	2.9 (2.6 to 3.1)	2.5 (2.3 to 2.8)	2.6 (2.3 to 2.8)	2.5 (2.3 to 2.8)	2.3 (2.0 to 2.5)	−50 (−56 to −44)
Rural	9.6 (9.2 to 10.0)	10.1 (9.7 to 10.5)	10.6 (10.2 to 11.0)	9.4 (9.0 to 9.8)	8.8 (8.4 to 9.2)	8.3 (7.9 to 8.7)	7.9 (7.5 to 8.3)	6.9 (6.5 to 7.2)	6.4 (6.0 to 6.7)	6.1 (5.8 to 6.4)	5.1 (4.8 to 5.5)	−47 (−50 to −43)
**Age group**												
0–4 years	0.8 (0.4 to 1.1)	0.8 (0.4 to 1.1)	2.0 (1.4 to 2.6)	1.1 (0.7 to 1.5)	0.9 (0.5 to 1.3)	1.8 (1.3 to 2.4)	1.0 (0.5 to 1.4)	0.9 (0.5 to 1.3)	0.9 (0.5 to 1.3)	0.9 (0.5 to 1.3)	0.7 (0.4 to 1.1)	−5 (−52 to 91)
5–14 years	1.0 (0.7 to 1.3)	1.0 (0.7 to 1.3)	1.0 (0.7 to 1.2)	0.7 (0.5 to 1.0)	0.9 (0.6 to 1.2)	1.3 (0.9 to 1.6)	1.1 (0.8 to 1.5)	0.8 (0.5 to 1.0)	0.6 (0.4 to 0.8)	0.4 (0.2 to 0.6)	0.4 (0.2 to 0.6)	−59 (−77 to −27)
15–24 years	3.7 (3.2 to 4.2)	3.7 (3.2 to 4.1)	4.4 (3.9 to 4.9)	3.6 (3.2 to 4.1)	4.3 (3.8 to 4.8)	3.5 (3.0 to 4.0)	3.0 (2.5 to 3.4)	2.4 (2.0 to 2.7)	2.0 (1.7 to 2.4)	1.7 (1.4 to 2.0)	1.1 (0.9 to 1.4)	−69 (−76 to −60)
25–44 years	6.4 (6.0 to 6.8)	6.5 (6.0 to 6.9)	6.7 (6.2 to 7.1)	6.2 (5.8 to 6.6)	5.1 (4.7 to 5.5)	4.9 (4.5 to 5.3)	4.1 (3.8 to 4.5)	3.9 (3.5 to 4.2)	3.2 (2.9 to 3.5)	3.1 (2.8 to 3.4)	2.5 (2.2 to 2.8)	−61 (−66 to −56)
45–64 years	8.6 (8.0 to 9.2)	8.7 (8.1 to 9.4)	9.0 (8.4 to 9.7)	7.5 (6.9 to 8.0)	7.3 (6.8 to 7.8)	6.5 (6.0 to 7.0)	6.9 (6.4 to 7.4)	5.5 (5.1 to 6.0)	5.0 (4.6 to 5.4)	5.1 (4.7 to 5.5)	5.0 (4.5 to 5.4)	−42 (−48 to −36)
≥ 65 years	27.3 (25.5 to 29.1)	28.0 (26.2 to 29.8)	28.6 (26.8 to 30.4)	27.0 (25.3 to 28.8)	25.6 (23.9 to 27.2)	20.9 (19.5 to 22.3)	20.6 (19.2 to 22.0)	18.6 (17.3 to 19.9)	19.8 (18.5 to 21.2)	18.9 (17.6 to 20.2)	16.0 (14.8 to 17.1)	−42 (−47 to −36)
**External cause**												
Drug^b^	0.6 (0.6 to 0.7)	0.7 (0.7 to 0.8)	0.8 (0.7 to 0.9)	0.6 (0.5 to 0.7)	0.6 (0.5 to 0.7)	0.5 (0.4 to 0.6)	0.5 (0.4 to 0.5)	0.4 (0.4 to 0.5)	0.4 (0.4 to 0.5)	0.5 (0.4 to 0.5)	0.3 (0.3 to 0.4)	−51 (−61 to −39)
Alcohol	0.1 (0.1 to 0.1)	0.1 (0.1 to 0.1)	0.1 (0.1 to 0.2)	0.1 (0.1 to 0.1)	0.1 (0.1 to 0.1)	0.1 (0.1 to 0.1)	0.1 (0.1 to 0.1)	0.1 (0.0 to 0.1)	0.1 (0.0 to 0.1)	0.1 (0.1 to 0.1)	0.1 (0.1 to 0.1)	−7 (−42 to 50)
Pesticides	5.6 (5.4 to 5.9)	5.8 (5.5 to 6.0)	5.9 (5.6 to 6.1)	5.5 (5.2 to 5.7)	5.0 (4.8 to 5.2)	4.2 (4.0 to 4.4)	4.1 (3.9 to 4.3)	3.6 (3.4 to 3.8)	3.3 (3.1 to 3.4)	3.1 (2.9 to 3.3)	2.6 (2.5 to 2.8)	−54 (−57 to −50)
Other gases/vapours	0.7 (0.6 to 0.8)	0.6 (0.6 to 0.7)	0.9 (0.8 to 1.0)	0.8 (0.7 to 0.8)	0.8 (0.7 to 0.9)	0.9 (0.8 to 1.0)	0.8 (0.7 to 0.9)	0.7 (0.6 to 0.7)	0.7 (0.6 to 0.8)	0.6 (0.6 to 0.7)	0.7 (0.6 to 0.8)	−1 (−16 to 17)
Other poisoning	0.5 (0.5 to 0.6)	0.5 (0.4 to 0.6)	0.4 (0.3 to 0.4)	0.3 (0.2 to 0.4)	0.3 (0.2 to 0.3)	0.3 (0.2 to 0.4)	0.3 (0.2 to 0.3)	0.2 (0.1 to 0.2)	0.2 (0.1 to 0.2)	0.2 (0.1 to 0.2)	0.1 (0.1 to 0.1)	−80 (−86 to −73)
**Intent**												
Unintentional	1.4 (1.3 to 1.6)	1.5 (1.4 to 1.6)	1.9 (1.7 to 2.0)	1.8 (1.6 to 1.9)	1.8 (1.7 to 1.9)	1.8 (1.7 to 1.9)	1.7 (1.6 to 1.8)	1.4 (1.3 to 1.5)	1.4 (1.3 to 1.5)	1.4 (1.3 to 1.5)	1.3 (1.1 to 1.4)	−12 (−22 to −1)
Suicide	5.8 (5.6 to 6.1)	6.1 (5.8 to 6.3)	6.0 (5.7 to 6.2)	5.3 (5.0 to 5.5)	4.8 (4.6 to 5.0)	4.1 (3.9 to 4.3)	3.9 (3.7 to 4.1)	3.4 (3.2 to 3.6)	3.1 (2.9 to 3.3)	3.0 (2.8 to 3.2)	2.5 (2.3 to 2.6)	−57 (−60 to −54)
Undetermined	0.3 (0.2 to 0.3)	0.2 (0.1 to 0.2)	0.3 (0.2 to 0.3)	0.2 (0.1 to 0.2)	0.1 (0.1 to 0.2)	0.1 (0.1 to 0.2)	0.1 (0.1 to 0.1)	0.1 (0.1 to 0.1)	0.1 (0.0 to 0.1)	0.1 (0.1 to 0.1)	0.1 (0.1 to 0.1)	−74 (−83 to −62)

CI: confidence interval.

^a^ Percentage change in mortality and its 95% CI was calculated as: (mortality rate ratio−1) × 100.

^b^ Drugs include nonopioid analgesic, antipyretic and antirheumatic drugs; sedative-hypnotic, antiparkinsonism and psychotropic drugs and narcotics and psychodysleptics [hallucinogens].

Notes: Mortality for overall and subgroup poisoning rates (excluding the six age groups) were age-adjusted using the population of China in 2010 (the most recent census).

Across the study time period, poisoning mortality in rural areas was 2.1–2.8 times greater than in urban areas. Male poisoning mortality per 100 000 people decreased over 2006‒2016 by 30% (from 13.7 to 9.6) for rural and 38% (from 6.3 to 3.9) for urban residents. Female poisoning mortality per 100 000 decreased by 47% (from 9.6 to 5.1) for rural and 50% (from 4.6 to 2.3) for urban residents.

Poisoning mortality generally rose as age increased. Substantial reductions in mortality occurred over 2006‒2016 in all sex- and age-specific groups except for children aged 0–4 years.

Among males, pesticides and alcohol were the most common substances involved, accounting for 48% (1516 of 3175) and 22% (711 of 3175) of poisoning deaths in 2016, respectively. Deaths per 100 000 people due to poisoning by pesticides and alcohol among males decreased by 44% (from 5.9 to 3.3) and 20% (from 2.0 to 1.6) respectively over the study period. Among females, pesticides were the most commonly used substances in poisoning deaths, accounting for  1209 of 1761 (69%) deaths in 2016. Deaths from poisoning by pesticide among females decreased by 54% (from 5.6 to 2.6 per 100 000) between 2006 and 2016. 

Suicidal poisoning mortality per 100 000 people decreased by 50% (from 6.3 to 3.1) in males and 57% (from 5.8 to 2.5) in females. This contrasts with a non-significant decrease in unintentional fatal poisonings in males (5%, from 3.8 to 3.6) and in females (7%, from 1.4 to 1.3).

### Location- and sex-specific trends

Analysis by location showed that mortality in all subgroups by intent and by type of substance were higher in rural than urban areas. Similar patterns of change over time were observed for urban and rural areas (data available from the corresponding author).

Sex-specific analysis demonstrated that males had higher subgroup poisoning mortality (by intent and by type of substance) than females, particularly for alcohol-related poisoning (data available from the corresponding author). The patterns of change from 2006 to 2016 by intent and by type of substance were generally similar among males and females.

### Age-specific trends

**Fig. 1:**
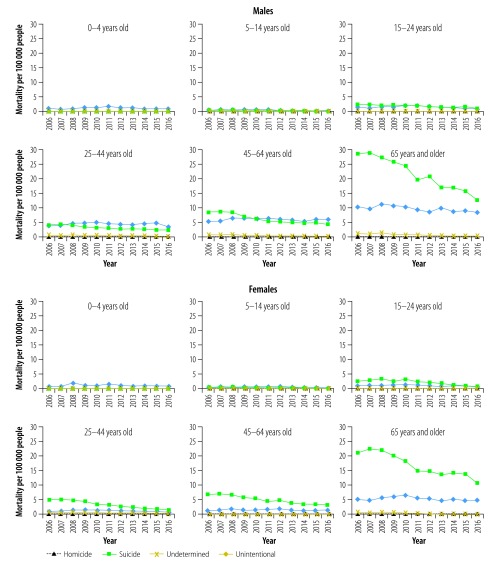
Age-adjusted poisoning mortality by age group and intent in China, 2006–2016

Note: Mortality for overall and subgroup poisoning rates were age-adjusted using the population of China in 2010.

Subgroup analyses by age group and intent show great variations across age groups (Fig. 1). Unintentional poisoning was the leading intent in children younger than 5 years, accounting for 40 of 42 (95%) poisonings for both sexes in 2016. Suicidal poisoning was the leading cause of fatal poisonings in individuals older than 24 years, and was especially prominent in the oldest individuals, constituting 59% and 68% of poisonings in males and females aged ≥ 65 years in 2016. Suicide poisoning mortality decreased substantially in most age groups for both males and females (data available from the corresponding author).

**Fig. 2:**
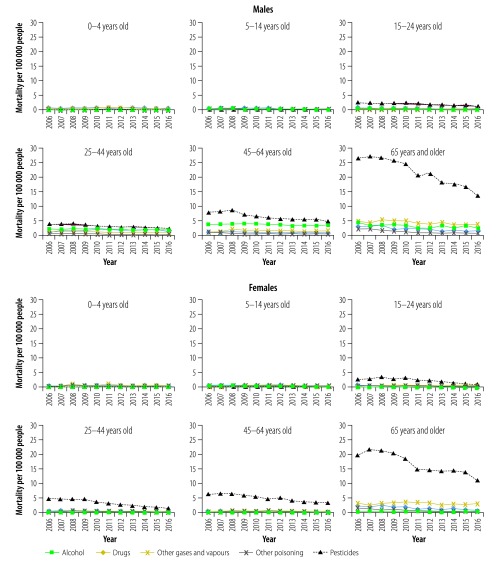
Age-adjusted poisoning mortality by age group and type of substance in China, 2006–2016

Notes: Mortality for overall and subgroup poisoning rates were age-adjusted using the population of China in 2010. Drugs include nonopioid analgesic, antipyretic and antirheumatic drugs; sedative-hypnotic, antiparkinsonism and psychotropic drugs and narcotics and psychodysleptics [hallucinogens].

Subgroup analyses by age group and type of substance demonstrated inconsistent changes over time from 2006 to 2016 (Fig. 2). For both sexes, pesticides, other gases and vapours, and drugs were the most common substances causing poisoning in children younger than 5 years. Among all other age groups, pesticide poisoning was most common. Alcohol poisoning deaths also occurred frequently among males aged 25–44 years and 45–64 years, accounting for 203 of 796 (26%) and 397 of 1254 (32%) deaths in 2016, respectively.

Analysis of changes over time found that decreases in pesticide poisoning mortality ranged from 38% to 66% for males and from 43% to 72% for females in all adolescent and adult age groups. Drug poisoning mortality per 100 000 people decreased by 11% (from 0.9 to 0.8) and 50% (from 2.8 to 1.4) in males and 43% (from 0.7 to 0.4) and 63% (from 2.4 to 0.9) in females in age group 45–64 years and ≥ 65 years respectively. Alcohol poisoning decreased in two male age groups: 25–44 years (from 2.2 to 1.6) and ≥ 65 years (from 4.2 to 2.3; data available from the corresponding author).

**Fig. 3:**
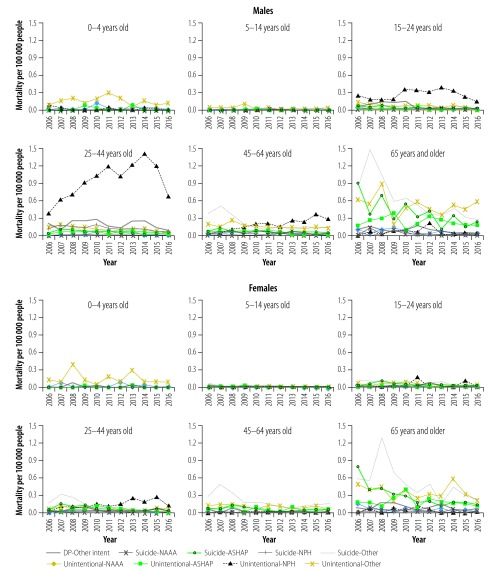
Age-adjusted, drug-induced poisoning mortality by intent and type of drug in China, 2006–2016

NAAA: nonopioid analgesic, antipyretic and antirheumatic drugs; ASHAP: sedative-hypnotic, antiparkinsonism and psychotropic drugs; NPH: narcotics and psychodysleptics [hallucinogens]; Other intent: drug poisoning with other intent.

Notes: All age- and drug-specific poisoning mortality rates were presented to show the changing pattern although most rates were unstable because of small numerators < 20 deaths. Mortality for overall and subgroup poisoning rates were age-adjusted using the population of China in 2010.

Subgroup analysis examining the combination of intent and type of drugs revealed various age-specific patterns in drug poisoning (Fig. 3). For both sexes, drug poisonings were rare in young children (Fig. 2). In 2016, for example, mortality per 100 000 in children was 0.17 for ages 0–4 years and 0.04 for ages 5–14 years. Deaths were primarily due to unintentional and suicidal poisonings by other drugs in age group 5–14 years. In males, unintentional poisoning by narcotics and psychodysleptics was the leading cause of drug poisoning for age groups 15–24 years and 25–44 years. Unintentional poisoning by narcotics and psychodysleptics or by other drugs and suicide poisoning by other drugs were most frequent in males aged 45–64 years and ≥ 65 years. Unintentional and suicide poisonings by other drugs were most common in females aged 45–64 years and ≥ 65 years. Changes in drug poisoning mortality varied greatly across sex- and age-specific groups (data are available from the corresponding author).

**Fig. 4:**
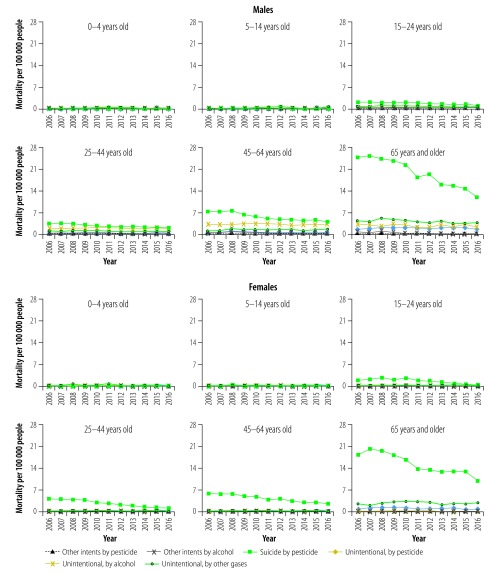
Poisoning mortality by age group, intent and type of substance (excluding drugs) in China, 2006–2016

Notes: Figures for age groups 0–4 years and 5–14 years were based on unstable mortality rates with numerators < 20 deaths. Mortality for overall and subgroup poisoning rates were age-adjusted using the population of China in 2010.

Subgroup analysis by both intent and type of substance revealed similar patterns of pesticide and alcohol poisoning deaths across the four adolescent and adult age groups (Fig. 4**)**. For both sexes, unintentional poisoning by pesticide and other gases and vapours were most common in age group 0–4 years. Poisoning mortality varied differently between subgroups over the study period. Despite small numbers, unintentional poisoning by alcohol increased 217% in females aged 45–64 years from 0.06 to 0.19 per 100 000 over 2006‒2016.

## Discussion

We generated five major findings. First, age-adjusted poisoning mortality dropped from 9.15 to 5.40 per 100 000 people between 2006 and 2016 in China. The reduction was present in both sexes and both urban and rural areas. Second, a reduction in suicidal poisoning by pesticides was the primary driver of recent decreases in overall poisoning mortality. Third, changes in overall poisoning mortality varied greatly across age groups in the analyses of particular intents and substances and patterns of change over time. Fourth, unintentional poisonings from alcohol represented a major subcategory of fatal poisonings for males ages 25–44 years and 45–64 years, and did not show downward trends in frequency over time. Fifth, despite the small numbers of deaths, large increases in unintentional drug poisoning by narcotics and psychodysleptics were found for individuals ages 25–44 years. We discuss each point below.

The study replicates the findings in two previous publications from China,^13^^,^^14^ and extends previous work. We found that the largest decreases in poisoning mortality in China occurred in unintentional poisoning and suicide by pesticide. The 2016 crude poisoning mortality in China (7.4 per 100 000 people in males and 4.3 in females) is lower than previous reports for northern European countries (ranging from 7.9 to 22.4 per 100 000 people)^6^^,^^8^ and the USA (16.2 in per 100 000 people).^2^ Variations across countries may reflect cultural differences, including societal reactions to suicide and suicide risk, management of lethal and poisonous substances, living and work environments and the reporting quality of poisoning mortality data (such as under-reporting due to the stigma attached to suicide).^24^^,^^25^

Consistent with previous studies both in China^14^ and elsewhere,^2^ our study reports higher poisoning mortality risk in males, rural residents and older adults. Higher risk among males could result from a combination of a greater tendency towards risk-taking and greater opportunity for exposure to poisoning, including being more likely to take on high-risk jobs and having higher alcohol consumption rates.^26^^,^^27^ Similarly, rural residents have greater access and exposure to poisonous substances such as pesticides. Greater fatality rates in rural areas of China may be also due to relatively underdeveloped prehospital aid and hospital treatment services for poisoning cases compared with those of urban cities.^28^ Older adults may face greater unintentional poisoning risk because of poorer physical health (such as weakened eyesight or sense of smell) and greater suicide risk from depressive disorders in older age.^1^

As reported in other Asian countries,^24^^,^^29^^,^^30^ suicide by pesticide was the most common cause of fatal poisonings in China. This may result primarily from easy access to pesticides that are used in agricultural production. Unlike the situation in high-income countries, where there is more mechanization and larger-scale farming, rural populations in low- and middle-income countries farm on small plots and have immediate access to pesticides.^31^

Encouragingly, suicidal poisoning by pesticides demonstrated a distinct decreasing trend from 2006 to 2016, which was an important component of the declining overall trend in suicides for China over that time period.^32^ The decrease likely reflects the effect of rapid urbanization that caused many citizens to migrate from rural to urban areas, reducing exposure and access to lethal pesticides.^33^^,^^34^ It may also reflect the impact of national changes in regulations on the production, circulation and sale of lethal pesticides.^35^^,^^36^ Social and economic development has improved living standards across both urban and rural China,^32^ reducing small-household farming practices. National development has also improved the capacity of mental health services,^37^ which may reduce suicides associated with chronic or acute mental illness. Older Chinese adults are less likely to migrate into urban areas and the number of older adults living in rural areas is actually increasing relative to previous years because of increased lifespans. Prevention efforts to reduce pesticide poisoning in rural China have been piloted, such as household lock-boxes and community education, but have so far been implemented in only a few areas.^38^

Poisoning deaths by pesticide and other gases and vapours among children aged 0–4 and 5–14 years remained relatively stable, a finding that deserves attention from researchers and policy-makers. Young children are full of curiosity, traits essential to learning and growth, but may lack the knowledge to judge the risks from lethal substances. Subgroup analysis by ICD-10 code showed that unintentional pesticide ingestions, children failing to recognize ingestion risks, was a major cause of unintentional poisoning by pesticide. Deaths from gases and vapours were primarily from carbon monoxide exposure. A study in Wuhan city reported multiple causes of carbon monoxide poisoning, including fire-related incidents, gas leaks, liquefied gas, gas-related poisoning while showering (from natural gas heaters) and coal or charcoal burning.^39^ Many of these risks can be prevented through established prevention strategies.^40^

Alcohol poisoning declined steeply between 2006 and 2016 among Chinese men older than 65 years. This success is probably related to prevention efforts to reduce harmful use of alcohol, including introduction of strict penalties for drink–driving since 2007^40^^,^^41^ and advocating moderate drinking as a component of healthy lifestyles.^42^

Between 2006 and 2016, we observed overall increases in unintentional drug poisoning by narcotics and hallucinogens among Chinese men aged 25–64 years and Chinese women aged 25–44 years, a trend that replicates concerns elsewhere in the world.^43^^,^^44^ These results may indicate the growing use of narcotics and psychodysleptics through both legal and illegal channels to treat pain and other conditions, as well as the rising production of fentanyl in China. All-age prevalence rates increased from 112.5 to 124.2 per 1000 persons for low back neck pain and from 0.76 to 0.80 per 1000 persons for drug use disorders between 2005 and 2015 in China.^1^ China is a primary producer of non-pharmaceutical fentanyl,^45^ creating potential for easy access among Chinese citizens.

Our analyses were limited by the lack of data on non-fatal poisoning and relevant risk factors such as access to lethal poisonous substances for vulnerable children and older adults, sale of prescription drugs, alcohol drinking behaviours and enforcement of relevant laws on pesticides, alcohol and prescription drugs. Without these data, causal inferences cannot be made. Additionally, our results are affected by the reporting quality of the disease surveillance data, including completeness, validity of classification and stability. A previous study reported that a small proportion of suicide deaths (5%), including those by poisoning, were misclassified as other specified injuries or injuries with unknown external causes.^46^ In addition, the introduction of a web-based reporting system in 2008 caused a slight fluctuation in reported mortality rates in the disease surveillance data set around 2008.^47^

Our findings have at least two policy implications. First, the findings offer evidence that some recent poisoning prevention policies and interventions may have achieved success. For example, new policies related to pesticide production, sale and storage may have contributed to a reduction in suicidal poisoning from pesticides. Similarly, there have been efforts to reduce harmful use of alcohol,^42^ including efforts to alter traditional attitudes of drinking alcohol as a way to enhance social connections,^26^ efforts to standardize and manage advertising of alcoholic beverages in the mass media,^48^ and the introduction of more severe drinking and driving laws.^49^ These may have contributed to a reduction in fatal alcohol poisoning incidents. Policy efforts should be continued.

Second, we noted large increases in unintentional drug poisoning from narcotics and hallucinogens in Chinese citizens aged 25–64 years, forewarning the risk of a national epidemic if strong actions are not taken. National efforts to continue to prohibit illegal production and sale of non-pharmaceutical fentanyl are needed.
